# Cocaine self-administration in rats lacking a functional *trpc4* gene

**DOI:** 10.12688/f1000research.2-110.v1

**Published:** 2013-04-17

**Authors:** Kristin C Rasmus, Casey E O'Neill, Ryan K Bachtell, Donald C Cooper

**Affiliations:** 1 Center for Neuroscience, Institute of Behavioral Genetics and Department of Psychology and Neuroscience, University of Colorado, Boulder, Boulder, CO, 80309, USA

## Abstract

The canonical transient receptor potential (TRPC) family of Ca ^2+^ permeable, non-selective cation channels is abundantly expressed throughout the brain, and plays a pivotal role in modulating cellular excitability. ****Unlike other TRPC channels, TRPC4 subtype expression in the adult rodent brain is restricted to a network of structures that receive dopaminergic innervation, suggesting an association with motivation- and reward-related behaviors. We hypothesized that these channels may play a critical role in dopamine-dependent drug-seeking behaviors. Here, we gathered data testing *trpc4* knockout (KO) rats and wild-type (WT) littermates in the acquisition of a natural sucrose reward (10 days), and cocaine self-administration (13 days) at 0.5 mg/kg/infusion. Rats lacking the *trpc4* gene ( *trpc4*-KO) learned to lever press for sucrose to a similar degree as their WT controls. However, when they were switched to cocaine, the *trpc4-*KO rats had substantially reduced cocaine-paired lever pressing compared to WT controls. No obvious group differences in inactive lever pressing were observed, for any time, during cocaine self-administration.

## Introduction

Canonical transient receptor potential (TRPC) channels are a group of non-selective cation channels that have recently gained more attention due to their involvement in neuronal excitability ^1^. This family of channels consists of 7 members (TRPC1-7) that can be turned on in response to the activation of the Gq alpha subunit of G-protein-coupled receptors ^2^. Stimulation of Gq alpha protein-coupled receptors activates phospholipase C Beta (PLCβ) producing elevations in inositol triphosphate (IP _3_) and intracellular Ca ^2+^ ^3^. TRPC channels contain three calmodulin sites and an IP _3_ site on the C-terminus of each subunit ^4, 5^. Thus, intracellular signaling resulting from Gq alpha protein-coupled receptors can enhance the activity of TRPC channels ^2^. These unique properties allow these channels to play a pivotal role in responding to intracellular Ca ^2+^ signaling, thereby affecting neuronal excitability. The TRPC4 channel is one of the two most abundant TRPC channel subtypes found in the adult mammalian brain ^6^. We have shown that TRPC4 channels are highly expressed in corticolimbic regions including the lateral septum, hippocampus, prefrontal cortex (PFC), and the amygdala ^6^. The expression pattern of the TRPC4 channels within brain reward areas along with its ability to regulate neuronal excitability raised the interesting possibility that TRPC4 channels are important for motivated behaviors and may play a role in drug reinforcement. In the present study, we explored cocaine self-administration in *trpc4* KO rats and their WT littermates to test the hypothesis that TRPC4 channels play a role in natural (sucrose) and drug (cocaine) reward-related behaviors.

## Materials and methods

### Animals

All experiments were conducted in accordance with guidelines of the ‘Institutional Animal Care and Use Committees’ at the University of Colorado at Boulder. Eight-week-old (250–300 grams) male *trpc4*-KO rats and their wild-type Fischer 344 littermates (Transposagen, Lexington, KY, USA) in these studies were housed separately. The *trpc4*-KO rats were generated using the Sleeping Beauty transposon system ^6^. We have previously reported some phenotypic characteristics of *trpc4*-KO rats that indicate a reduction in social interaction and normal learning on simple and complex tasks ^7– 9^. In the sucrose self-administration studies, nine *trpc4*-KO, nine WT and 7 heterozygote rats were used, while 7 animals from each strain were used in all cocaine self-administration experiments.

### Animal genotyping and PCR

Three polymerase chain reaction (PCR) primers were designed and used as 20–24 oligonucleotide sequences (Eurofins MWG Operon, Ebersberg, Germany) to genotype rats. Reactions were carried out using Choice-Taq Blue DNA polymerase (Denville Scientific Inc., Metuchen, NJ, USA) in a Techne Touchgene thermal cycler (Techne, Minneapolis, MN, USA). Ethidium bromide-stained 1.5% agarose gels (Thermo Fisher Scientific Inc., Pittsburgh, PA, USA) were photographed with a Kodak Gel Logic 200 UV transilluminator imager (Carestream Health Inc., Rochester, NY, USA) using a 100 bp DNA ladder in the first lane of the gel as reference (New England BioLabs, Ipswich, MA, USA).

### Sucrose self-administration

All self-administration procedures were performed in operant conditioning chambers (Med-Associates, St. Albans, VT, USA) equipped with two response levers, a sucrose hopper, and an infusion pump system. Animals were food-deprived to 85% free feeding weight and trained to lever-press for sucrose pellets on a fixed ratio 1 (FR1) reinforcement schedule for 5 days/week. A discriminative cue light stimulus was illuminated above the lever paired with sucrose delivery. A correct lever response resulted in the delivery of a sucrose pellet (45 mg). With the termination of the cue light and a 20 second time out period (TO 20s), responding produced no consequence. Inactive lever responses produced no consequence throughout the session. The session was complete when the animal had administered 50 sucrose pellets and the latency (in minutes) to acquire 50 pellets was recorded as the dependent variable. Failure to reach criteria (50 pellets) within 120 minutes resulted in termination of the session. Animals completed 10 sucrose self-administration sessions, which was sufficient to establish stable baseline sucrose responding in all groups.

### Surgery

Following sucrose self-administration, rats were given *ad libitum* food. After 24–48 hours of free feeding, catheters were implanted into the jugular vein under halothane anesthesia (1–2.5%). During recovery, catheters were flushed daily with 0.1 ml of 0.9% heparinized saline to maintain patency. Rats were allowed 4–7 days to recover in their home cage before experimental procedures began.

### Cocaine self-administration procedure

Following at least 4–7 days recovery from surgery, animals were trained to self-administer intravenous cocaine (0.5 mg/kg/100 μl injection) on an FR1 schedule in daily 2-hour sessions for 5 days/week. Cocaine injections were delivered over 5 seconds and were concurrent with the illumination of a cue light above the active lever. Drug and cue delivery were followed by a 15 second time out period, where the house light remained off and responding produced no consequence. Inactive lever responses produced no consequence throughout testing. No differences were observed between groups on the inactive lever (see data set).

### Catheter verification

Catheter patency was tested at the completion of the experiment to ensure that differences in self-administration were not due to catheter failure. Fatal Plus cocktail (390 mg/ml pentobarbital sodium, 0.01 mg/ml propylene glycol, 0.29 mg/ml ethyl alcohol, 0.2 mg/ml benzyl alcohol) was administered through the animal’s catheter at 0.2 ml/kg. Catheter patency was confirmed if the animal responded immediately (within 1 second) with muscle atonia and lethality following Fatal Plus administration. Animals with faulty catheters (n=4) were excluded from these studies (see data set).

### Drugs

Fatal Plus was obtained from Vortech Pharmaceuticals, LTD (Dearborn, MI, USA). Cocaine hydrochloride was obtained from Sigma-Aldrich (St. Louis, MO, USA). Cocaine was dissolved in sterile-filtered physiological saline (0.9%).

## Results

### Generation of *trpc4* knock-out rats

**Figure 1.**
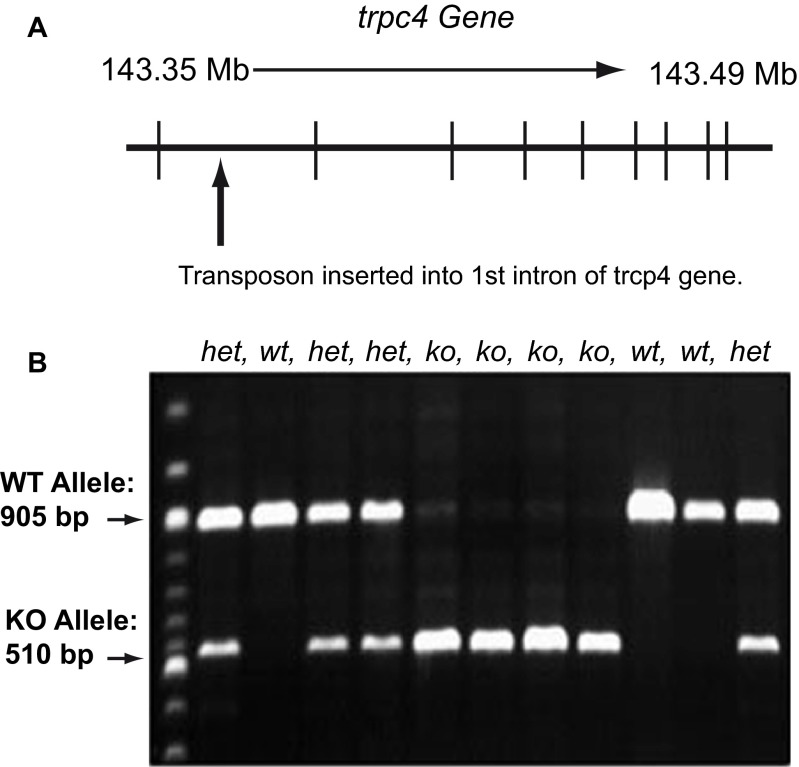
Generation of the *trpc4* knock-out (KO). **A**. Schema of the *trpc4* gene in the rat genome and the Sleeping Beauty (SB) gene knock-out system. The *trpc4* gene is located on chromosome 2 of the rat genome, between 143.35 Mb and 143.49 Mb. The SB transposon was inserted into the first intron of *trpc4*, therefore creating a complete knock-out of the coding sequence. **B**. Ethidium bromide-stained agarose gel visualizing the 905 bp marker for the wild-type allele and the 510 bp marker for the *trpc4* KO allele. To genotype the animals, 1.5% agarose gel electrophoresis was used.

The Sleeping Beauty (SB) gene-trap transposon method was used to create the *trpc4*-KO animals ^7^. The SB method uses cut-and-paste transposable elements to generate heritable loss-of-function mutations. Figure 1A shows the location of the *trpc4* gene on the rat genome and where the transposon was inserted. By inserting the SB transposon into the first intron of the *trpc4* gene, the full-length protein product can be deleted. Using primers for the *trpc4*-KO and WT alleles, we were able to confirm the deletion using PCR and gel electrophoresis ( Figure 1B).

### Sucrose self-administration

**Figure 2.**
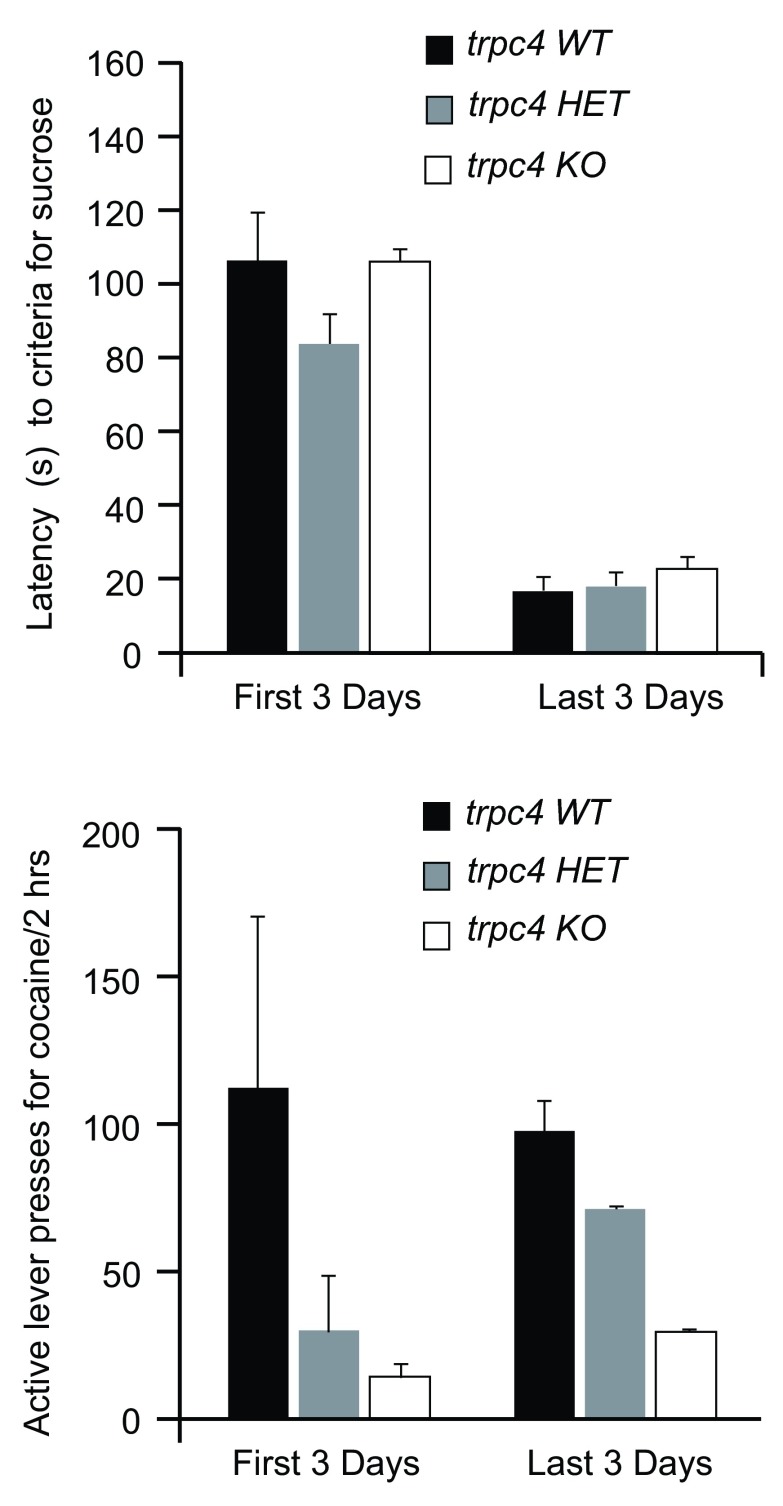
Sucrose and cocaine self-administration in the *trpc4*-KO, WT and HET rats. **A**) The average latency in seconds for the *trpc4* knock-out (KO), wild-type (WT) and heterozygote (HET) rats to acquire 50 sucrose pellets in the allotted time period (120 minutes) during the first 3 days and the last 3 days is shown. The animals learned to lever-press for sucrose pellets on a fixed-ratio 1 (FR1) reinforcement schedule for 5 days/week. All self-administration chambers were equipped with an active and inactive lever. The active lever was paired with a discriminative cue light stimulus. A correct lever response resulted in the sucrose reward, the termination of the cue light, and a 20 second time-out (TO 20s) period where responding produced no consequences. **B**) The average self-administered cocaine for WT, HET and KO rats during daily 2 hour sessions on a FR1 reinforcement schedule for the first 3 days and last 3 days of acquisition is shown.

As shown in Figure 2A, both the *trpc4*-KO rats and their WT littermates acquired 50 sucrose pellets at similar levels during the first 3 days and last 3 days of sucrose training. By day 7, all groups reached stable baseline, responding averaged between 16 and 26 seconds to acquire 50 pellets. Thus, it appears that *trpc4*-KOs do not differ in their ability to learn to self-administer a natural reward, sucrose, on a FR-1 schedule (see data set). The submitted data set contains responding on the active and inactive levers and the number of sucrose pellets for all three treatment groups across ten days of sucrose training.

### Cocaine self-administration

We next sought to determine whether *trpc4*-KO rats would differ in cocaine reinforced behavior. Cocaine self-administration summary results are presented in the *trpc4*-KO, WT and *trpc4* heterozygote (HET) rats on an FR1 schedule of reinforcement ( Figure 2). Figure 2B illustrates a reduction in responding to cocaine in the *trpc4*-KO group over the first 3 days and last 3 days ( Figure 2B). The submitted data set contains responding on the active and inactive levers and the number of cocaine infusions for all three treatment groups across thirteen days of cocaine self-administration training.

## Summary

Data is presented showing acquisition of sucrose and cocaine self-administration infusions in *trpc4* WT, HET and KO rats. The summary data show that for the first and last 3 days, rats lacking a functional *trpc4* gene have normal sucrose mediated reward, and reduced acquisition of cocaine self-administration compared to WT rats.

Sucrose and cocaine self-administration data set This data set includes raw data for sucrose intake and cocaine self-administration experiments for all three rat strains used in our study – Trpc4-KO, wild-type and heterozygous animals. The sucrose intake data is over a 10-day period, while the cocaine self-administration data is 13 days. Both data sets include averages for the first 3 days and last three days of experiments, also shown in Figure 2. Catheter patency tests and lever press data is also included. Catheter patency was tested and recorded daily. Lever press data shows the total lever presses (inactive and active) for each animal, which will differ from number of drug infusions due to the time-out period between infusions.

**Raw data: Sucrose and cocaine self-administration data set**

This data set includes raw data for sucrose intake and cocaine self-administration experiments for all three rat strains used in our study – *trpc4*-KO, wild-type and heterozygous animals. The sucrose intake data is over a 10-day period, while the cocaine self-administration data is 13 days. Both data sets include averages for the first 3 days and last three days of experiments, also shown in Figure 2. Catheter patency tests and lever press data is also included. Catheter patency was tested and recorded daily. Lever press data shows the total lever presses (inactive and active) for each animal, which will differ from number of drug infusions due to the time-out period between infusions.
