# Supplementary material for: Cocaine self-administration in rats lacking a functional trpc4 gene
Source: F1000Res. 2013 Apr 17;2:110. [Version 1] doi: 10.12688/f1000research.2-110.v1 (PMC3901450; doi:10.12688/f1000research.2-110.v1)
Supplement: Peer review history

# Peer review: Referee response for version 1

**Published:** 2013-05-14

**Authors:** Eleanor Simpson

**Affiliations:** 1 Department of Psychiatry, Columbia University, New York State Psychiatric Institute, New York, NY, USA

This article addresses if loss of TRPC4 affects self administration of sucrose and cocaine. The title is appropriate and the abstract is a suitable summary of the article. The article is clearly written, the experiments are properly conducted and the methods are sufficiently described. The data file provided is clearly laid out, and most of the relevant data is included. It would be useful for the authors to also include lever press data for the sucrose self administration task, in case there is a strain difference in responding on the active lever during the 20s timeout periods between trials. 

Statistical analysis of both lever press data as well as # cocaine infusions across sessions will determine if the authors claim that acquisition of cocaine self administration is reduced in TRPC4 KO rats is statistically significant.

I have read this submission. I believe that I have an appropriate level of expertise to confirm that it is of an acceptable scientific standard.

# Peer review: Referee response for version 1

**Published:** 2013-05-10

**Authors:** Xiu-Ti Hu

**Affiliations:** 1 Department of Pharmacology, Rush University Medical Center, Chicago, IL, USA

The purpose of this study was to determine whether cocaine self-administration of rats was altered by *trpc4* knockout (KO). The title is appropriate and the Abstract is a suitable summary for this article. The article is well-constructed and clear. Outcomes from this study were important, suggesting a potential role of TRPC4 channels in neurons that mediate cocaine self-administration in the related structures of the rat brain. The study was well-designed and sensible data are obtained using a cutting-edge technology. Providing the methods and statistical results will further strengthen this article.

I have read this submission. I believe that I have an appropriate level of expertise to confirm that it is of an acceptable scientific standard.
